# Assessment of surgical sutures POLYMED^®^ by intracutaneous irritation test in rabbits

**DOI:** 10.2478/intox-2013-0017

**Published:** 2013-06

**Authors:** Rumyana Simeonova, Nikolai Danchev

**Affiliations:** Medical University, Faculty of Pharmacy, Department of Pharmacology, Pharmacotherapy and Toxicology, Sofia, Bulgaria

**Keywords:** surgical sutures, intracutaneous irritation test, rabbit

## Abstract

The aim of the present study was to evaluate local irritant effects to rabbit skin following a single application of test samples of non-sterile polyamide non-absorbable surgical sutures POLYMED^®^. The polar and nonpolar extracts were prepared by using saline solution and olive oil, respectively, after sinking the materials tested (2.0 g) in 10 ml of the corresponding liquid. Incubation was carried out at the temperature of 37 °C for 72 h. The saline solution and pure olive oil, which had no contact with the materials tested, were used as negative control samples and were incubated under the same conditions as above. Assessments of the extracts from each material were conducted on 2 albino rabbits of the New Zealand breed. On the back of each animal, 5 intracutaneous injections of the extract tested and 5 injections of the control solution, each of 0.2 ml, were carried out. The degree of irritation was scored at 4, 24, 48, 72 hours after injection and no skin changes were found. The intracutaneous irritation index (III) was calculated and yielded 0.0. Hence it was concluded that under the experimental conditions the extracts of the material tested, i.e. non-sterile polyamide non-absorbable surgical sutures POLYMED^®^, were ‘non-irritant’ to the skin of rabbits when compared with the respective control groups. The experimental procedure was conducted according to ISO10993-10.

## Introduction

POLYMED^®^ are non-sterile, synthetic, woven multifilament surgical sutures made of Polyamide 6/6 (Medica webpage - http://fw.medica.bg/en/home.html?products/surgicals.html). Surgical suture is a medical device used to hold body tissues together after an injury or surgery. As a medical device, surgical suture needs to be evaluated in terms of the possibility to cause irritation reaction or toxic response.

To minimize any potential hazards to the patients, it is essential that biocompatibility assessments be conducted for all materials that are used in medical devices. Depending on the application, no single test may be sufficient to define biocompatibility. Thus multiple tests may be needed to determine the biocompatibility of the material. Two common tests are used to measure biocompatibility, USP 31. NF 26 (2008), Biological Reactivity Tests *in vivo*, and ISO 10993, Biological Evaluation of Medical Devices (2006; 2007).

The present experiment was conducted according to the method of examination of intracutaneous reactivity ISO 10993-10 (2006). The ISO intracutaneous reactivity test is the standard protocol for determining a medical device‘s potential for causing irritation. Intracutaneous (intradermal) reactivity tests determine the localized reaction of tissue to extracts of medical devices, biomaterials, or prostheses in the final product form. Irritation and intracutaneous tests may be applicable where determination of irritation by dermal or mucosal irritation tests is not appropriate. Albino rabbits are most commonly used. Because these tests focus on determining the biological response of leachable agents that may be present in biomaterials, their extracts in various solvents are utilized to prepare the injection solutions. Critical to the conduct of these tests is the preparation of the test material and/or extract solution and the choice of solvents, which must have physiological relevance (Buddy *et al.*, 2004). The method appears in many national compendial references, such as the US Pharmacopeia (USP 31. NF 26, 2008).

## Materials and methods

Water bath shaker (Memmert - ELTA 90, Bulgaria), Physiological Saline (Sopharma, Bulgaria), Olive Oil (Sigma, Germany), 25G Needle, Syringe (Momina krepost, Bulgaria) were used for the study.

### Animals

In the present study, two adult male rabbits (applying 3R principle according to Upman *et al.* (2003) and Robinson (2005)) of the New Zealand White Strain, weighing not less than 2 kg, were used. The animals were purchased from the National Breeding Centre, Slivnitza, Bulgaria. The rabbits were housed isolated in metal cages (50×50×55 cm) fitted with perforated floors under standard laboratory conditions (ambient temperature 20±2 °C and humidity 72±4%) with free access to water and standard pelleted rabbit food 53-3, produced according to ISO 9001 (2008). A minimum of 7 days of acclimatization was allowed before the commencement of the study and their health was monitored regularly by a veterinary physician, according to the requirements of ISO 10993-10 (2006). Vivarium (certificate of registration of farm No. 0072/01.08.2007) was inspected by the Bulgarian Drug Agency in order to check the husbandry conditions (No. A-11-1081/03.11.2011). All procedures performed were approved by the Institutional Animal Care Committee and the principles stated in the European Convention for the Protection of Vertebrate Animals Used for Experimental and Other Scientific Purposes (ETS 123) (Council of Europe, 1991) were strictly followed throughout the experiment. Each rabbit cage was attached with a tag marked with the animal number, the test and the product name.

### Preparation of the test material extracts (ISO 10993-12)

Two grams of the test material (surgical sutures POLYMED^®^) was extracted in 10 ml of physiological saline and in 10 ml of olive oil. The elution was performed by gentle shaking (100 rpm) in a water bath shaker at 37 °C for 72 hours. Saline solution and pure olive oil which had no contact with the materials tested were used as negative control samples and were incubated under the same conditions as above. The flasks with extraction medium and test material were closed with a parafilm during the whole period of extraction. The extracts were used within 4 hours to perform the test procedure.

### Intracutaneous irritation test (ISO 10993-10)

On the day before the experiment, the fur on the back of the animals was closely clipped on both sides of the spine. There were no mechanical irritation and lesions in the skin. On the day of the experiment, the skin was cleaned with 70% ethanol and dried. The surgical suture material extracted in physiological saline and olive oil was aseptically injected intracutaneously (i.c.) at a dose of 0.2 ml into five sites on the upper left hand side and right hand side of the two rabbits. The physiological saline alone and olive oil alone (control extract) were injected at the same dose of 0.2 ml into five sites on the lower-left hand side and lower-right hand side of the same rabbits. Signs of punctures appeared immediately after application and served as benchmarks for monitoring the animals. Skin reaction (erythema and/or edema), at the site of application was subjectively assessed and scored at 4, 24, 48, 72 hours after intracutaneous injection of the test materials (post test observation period). During the observation period, the animals were handled with care to avoid touching the injection sites. The reaction at the site of injections was assessed and scored according to the following numerical system:

**Table:** 

**(A) Erythema and eschar formation**	**Score**
No erythema	0
Very slight erythema (barely perceptible)	1
Well defined erythema	2
Moderate to severe erythema	3
Severe erythema (beet redness to eschar formation)	4
**(B) Edema formation**	
No edema	0
Very slight edema (barely perceptible)	1
Slight edema (edges of area well raised)	2
Moderate edema (edges raised 1 mm)	3
Severe edema (raised <1 mm and extending beyond area of exposure)	4
**Total possible score for dermal inflammation (irr.)**	**8**

### Evaluation of intracutaneous irritation index (III)

For each rabbit the irritation scores for both erythema and edema were added separately for each test extract at each observation point (4, 24, 48 and 72h) and were divided by the total number of observations. The scores for each animal were added and divided with the total number of animals. This index is the Intracutaneous Irritation Index (III).

**Table:** 

Evaluations	Mean score (III)
Non irritant	0.0
Negligible irritant	0.1–0.4
Slight irrritant	0.5–1.9
Moderate irritant	2.0–4.9
Severe irritant	5.0–8.0

## Results

Tables 1–4 show the irritation potential of the physiological saline and olive oil extracts of surgical sutures POLYMED^®^. It was found that the animals did not show any grade of erythema and/ or edema after intradermal injection of the material extracts studied. The average irritation score induced by the physiological saline and olive oil extract of surgical sutures was 0.0. The irritation potential induced by either material extracts was comparable to controls.

**Table 1:** Irritation effects of intracutaneous (i.c.) administration of polar (0.9% NaCl) extract (0.2 ml) of surgical sutures POLYMED^®^ in rabbit No. 1 compared with control.

Rabbit No. 1		Test material sites							Control sites				

Time	Reaction	1	2	3	4	5	Mean score	Combined index	1	2	3	4	5
4 h	Erythema	0	0	0	0	0	0.0		0	0	0	0	0
	Edema	0	0	0	0	0	0.0	0.0	0	0	0	0	0
24 h	Erythema	0	0	0	0	0	0.0		0	0	0	0	0
	Edema	0	0	0	0	0	0.0	0.0	0	0	0	0	0
48 h	Erythema	0	0	0	0	0	0.0		0	0	0	0	0
	Edema	0	0	0	0	0	0.0	0.0	0	0	0	0	0
72 h	Erythema	0	0	0	0	0	0.0		0	0	0	0	0
	Edema	0	0	0	0	0	0.0	0.0	0	0	0	0	0
Intracutan. Irrrit. Index (III)									0.0				

**Table 2:** Irritation effects of intracutaneous (i.c.) administration of polar (0.9 % NaCl) extract (0.2 ml) of surgical sutures POLYMED^®^ in rabbit No. 2 compared with control

Rabbit No. 2		Test material sites							Control sites				

Time	Reaction	1	2	3	4	5	Mean score	Combined index	1	2	3	4	5
4 h	Erythema	0	0	0	0	0	0.0		0	0	0	0	0
	Edema	0	0	0	0	0	0.0	0.0	0	0	0	0	0
24 h	Erythema	0	0	0	0	0	0.0		0	0	0	0	0
	Edema	0	0	0	0	0	0.0	0.0	0	0	0	0	0
48 h	Erythema	0	0	0	0	0	0.0		0	0	0	0	0
	Edema	0	0	0	0	0	0.0	0.0	0	0	0	0	0
72 h	Erythema	0	0	0	0	0	0.0		0	0	0	0	0
	Edema	0	0	0	0	0	0.0	0.0	0	0	0	0	0
Intracutan. Irrrit. Index (III)									0.0				

**Table 3:** Irritation effects of intracutaneous (i.c.) administration of non-polar (olive oil) extract (0.2 ml) of surgical sutures POLYMED^®^ in rabbit No. 1 compared with control.

Rabbit No. 1		Test material sites							Control sites				

Time	Reaction	1	2	3	4	5	Mean score	Combined index	1	2	3	4	5
4 h	Erythema	0	0	0	0	0	0.0		0	0	0	0	0
	Edema	0	0	0	0	0	0.0	0.0	0	0	0	0	0
24 h	Erythema	0	0	0	0	0	0.0		0	0	0	0	0
	Edema	0	0	0	0	0	0.0	0.0	0	0	0	0	0
48 h	Erythema	0	0	0	0	0	0.0		0	0	0	0	0
	Edema	0	0	0	0	0	0.0	0.0	0	0	0	0	0
72 h	Erythema	0	0	0	0	0	0.0		0	0	0	0	0
	Edema	0	0	0	0	0	0.0	0.0	0	0	0	0	0
Intracutan. Irrrit. Index (III)									0.0				

**Table 4:** Irritation effects of intracutaneous (i.c.) administration of non-polar (olive oil) extract (0.2 ml) of surgical sutures POLYMED^®^” in rabbit No. 2 compared with control.

Rabbit No. 2		Test material sites							Control sites				

Time	Reaction	1	2	3	4	5	Mean score	Combined index	1	2	3	4	5
4 h	Erythema	0	0	0	0	0	0.0		0	0	0	0	0
	Edema	0	0	0	0	0	0.0	0.0	0	0	0	0	0
24 h	Erythema	0	0	0	0	0	0.0		0	0	0	0	0
	Edema	0	0	0	0	0	0.0	0.0	0	0	0	0	0
48 h	Erythema	0	0	0	0	0	0.0		0	0	0	0	0
	Edema	0	0	0	0	0	0.0	0.0	0	0	0	0	0
72 h	Erythema	0	0	0	0	0	0.0		0	0	0	0	0
	Edema	0	0	0	0	0	0.0	0.0	0	0	0	0	0
Intracutan. Irrrit. Index (III)									0.0				

## Discussion

As a method for closing cutaneous wounds, the technique of suturing is thousands of years old. Sutures are natural or synthetic textile biomaterials widely used in wound closure, to ligate blood vessels and to draw tissues together. Sutures can be classified into two broad categories: absorbable and non-absorbable. In this study we used non-absorbable sutures. The most crucial requirements of sutures materials are physical and mechanical properties, handling properties, biocompatibility, and antimicrobial nature (Saxena *et al.*, 2011).

Biocompatibility is a general term used to describe the suitability of a material for exposure to the body or bodily fluids. Biocompatibility testing is essential for all materials that will be used in medical devices to minimize any potential hazards to the patient. A material will be considered biocompatible if it allows the body to function without any complications, such as allergic reactions, irritation or other adverse side effects. The present study can by considered as a part of the whole biocompatibility testing.

The U.S. Food and Drug Administration have adopted the ISO-10993 standard as its criteria for guiding the selection of biocompatibility testing for a given type of device. This should consist of *in vitro* and *in vivo* assessments that are relevant to the device application. No single test is sufficient to define biocompatibility and a variety of tests are necessary to determine biocompatibility, depending on the device and application.

In the present study an intracutaneous irritation test was performed according the ISO-10993-10 guideline (2006). The purpose of this testing was to determine the fitness of the surgical sutures POLYMED^®^ for human use and to see whether the use of this medical device can have any potentially harmful physiological effects as erythema and/or edema.

The result of the study showed that the material tested, non-sterile polyamide non-absorbable surgical sutures POLYMED^®^, extracted with non-polar and polar extraction solvent was found to be non-irritant (III=0.00) to the skin of the rabbits investigated within an observation period of 72 hours. The extracts tested showed the same irritation potential as the negative controls. This suggests that both the physiological saline and olive oil extract of the material tested met the requirements of the test since the difference between the test sample mean score and the control mean score was less than 1.0 (=0.00) as per ISO 10993-10 (2006) and declared as non-irritant.

It can be concluded that the test material non-sterile polyamide non-absorbable surgical sutures POLYMED^®^ did not cause an intracutaneous reaction under the described test conditions.
